# Synaptotagmin 11 scaffolds MKK7–JNK signaling process to promote stem-like molecular subtype gastric cancer oncogenesis

**DOI:** 10.1186/s13046-022-02420-3

**Published:** 2022-06-29

**Authors:** Bo-Kyung Kim, Da-Mi Kim, Hyunkyung Park, Seon-Kyu Kim, Mi-Aie Hwang, Jungwoon Lee, Mi-Jung Kang, Jae-Eun Byun, Joo-Young Im, Minho Kang, Kyung Chan Park, Young Il Yeom, Seon-Young Kim, Haiyoung Jung, Dae-Hyuk Kweon, Jae-Ho Cheong, Misun Won

**Affiliations:** 1 Personalized Genomic Medicine Research Center, KRIBB, 125 Kwahag-ro, Yuseong-gu, Daejeon, 34141 South Korea; 2 Present Address: KRIBB School of Bioscience, University of Science and Technology, Daejeon, South Korea; 3 R&D Center, oneCureGEN, Daejeon, South Korea; 4 Department of Integrative Biotechnology, College of Biotechnology and Bioengineering, Sungkyunkwan University, Suwon, South Korea; 5 Environmental Diseases Research Center, KRIBB, Daejeon, South Korea; 6 Immunotherapy Research Center, KRIBB, Daejeon, South Korea; 7 Korea Bioinformation Center, KRIBB, Daejeon, South Korea; 8 Department of Surgery, Yonsei University College of Medicine, Seoul, South Korea; 9 Serverance Biomedical Science Institute, Yonsei University College of Medicine, Seoul, South Korea

**Keywords:** GC, SYT11, Metastasis, Stem-like subtype, JNK

## Abstract

**Background:**

Identifying biomarkers related to the diagnosis and treatment of gastric cancer (GC) has not made significant progress due to the heterogeneity of tumors. Genes involved in histological classification and genetic correlation studies are essential to develop an appropriate treatment for GC.

**Methods:**

In vitro and in vivo lentiviral shRNA library screening was performed. The expression of Synaptotagmin (SYT11) in the tumor tissues of patients with GC was confirmed by performing Immunohistochemistry, and the correlation between the expression level and the patient’s survival rate was analyzed. Phospho-kinase array was performed to detect Jun N-terminal kinase (JNK) phosphorylation. SYT11, JNK, and MKK7 complex formation was confirmed by western blot and immunoprecipitation assays. We studied the effects of SYT11 on GC proliferation and metastasis, real-time cell image analysis, adhesion assay, invasion assay, spheroid formation, mouse xenograft assay, and liver metastasis.

**Results:**

SYT11 is highly expressed in the stem-like molecular subtype of GC in transcriptome analysis of 527 patients with GC. Moreover, SYT11 is a potential prognostic biomarker for histologically classified diffuse-type GC. SYT11 functions as a scaffold protein, binding both MKK7 and JNK1 signaling molecules that play a role in JNK1 phosphorylation. In turn, JNK activation leads to a signaling cascade resulting in cJun activation and expression of downstream genes angiopoietin-like 2 (ANGPTL2), thrombospondin 4 (THBS4), Vimentin, and junctional adhesion molecule 3 (JAM3), which play a role in epithelial-mesenchymal transition (EMT). SNU484 cells infected with SYT11 shRNA (shSYT11) exhibited reduced spheroid formation, mouse tumor formation, and liver metastasis, suggesting a pro-oncogenic role of SYT11. Furthermore, SYT11-antisense oligonucleotide (ASO) displayed antitumor activity in our mouse xenograft model and was conferred an anti-proliferative effect in SNU484 and MKN1 cells.

**Conclusion:**

SYT11 could be a potential therapeutic target as well as a prognostic biomarker in patients with diffuse-type GC, and SYT11-ASO could be used in therapeutic agent development for stem-like molecular subtype diffuse GC.

**Supplementary Information:**

The online version contains supplementary material available at 10.1186/s13046-022-02420-3.

## Background

Gastric cancer (GC) is among the most prevalent cancers globally, especially in Asia. Prognosis estimation and novel therapeutics development are difficult due to the tumor heterogeneity, the lack of biomarkers to definitively distinguish between the different GC types, and therapeutic target scarcity. Previous studies on GC genomic analysis by The Cancer Genome Atlas (TCGA) Research Network [1] and the Asian Cancer Research Group (ACRG) [2] have identified four distinct molecular subtypes linked to unique somatic alteration and clinical phenotype patterns: Epstein–Barr virus (EBV), microsatellite instability (MSI), chromosomal instability (CIN) and genomically stable (GS) subtypes by TCGA, and microsatellite instability (MSI), microsatellite stable with epithelial-mesenchymal transition (MSS/EMT), MSS/TP53+, and MSS/TP53- by ACRG [2]. The MSS/EMT subtype reportedly exhibits a particularly poor prognosis in the ACRG cohort. Recently, GC has been classified into more clinically relevant five molecular subtypes: inflammatory, intestinal, gastric, mixed stromal, and stem-like, each having different prognosis and therapeutic response to standard chemotherapy [3]. At the molecular level, HER2, EGFR, VEGF, SOX-9, c-MET, CEA, CA19–9 protein expression, and microRNAs have been proposed as potential GC diagnosis and prognosis biomarkers [4–6]. However, only HER2 inhibitors (e.g., trastuzumab) have been applied clinically. Given the clinical significance of GC molecular subtypes, additional candidate biomarkers are needed for novel therapeutic agent development, especially for the stem-like subtype. According to the Lauren histologic classification, GC is traditionally classified as intestinal- and diffuse-type [7]. Intestinal-type GC-derived cancer cells exhibit adhesion and a linear or tubular arrangement [8]. In contrast, diffuse-type GC-derived cancer cells lack adhesion, are not cohesive, and scatter easily to form metastases [8]. Diffuse-type GC mostly occurs in young women with poor prognosis compared to intestinal-type GC [8, 9].

In this study, we evaluated synaptotagmin 11 (SYT11) as a potential biomarker and therapeutic target for stem-like molecular subtype diffuse GC with worst prognosis. SYT11 is a synaptotagmins (SYTs) family member, which constitutes membrane trafficking proteins with two C-terminal C2 domains, an N-terminal transmembrane region, and a variable linker. Based on their sequences and properties, 15 SYTs are present in the mammalian family [10]. SYT11 has a conserved substitution of aspartate for a serine residue in the C2A domain that changes the Ca^2+^-binding properties of the C2A domain. Recently, SYT11 emerged as a new Parkinson’s disease-associated gene [11]. SYT11 is regulated at both the transcriptional and post-translational levels by ATP13A2, and ATP13A2-mediated autophagy responses are SYT11-dependent [11]. Several proteins reportedly interact with SYT11 in pancreatic β-cells [12]. The SYT11 C2B domain interacts with SND1, Ago2, and FMRP, components of the RNA-induced silencing complex [12]. The SYT family is involved in the gastric, lung, and colorectal cancer and gliomas cell proliferation and metastasis [13–19]. SYT7, SYT8, and SYT13, in particular, are reportedly associated with GC metastasis [14–16]. However, no study describes the cancer-related SYT11 functions and mechanisms.

Most cancers originate from epithelial tissues and EMT is associated with tumor initiation, progression, intravascular penetration into the bloodstream, migration, and metastasis, as well as resistance to therapy [20–22]. Invasive tumors exhibit a dedifferentiated morphology accompanied by a loss of epithelial or a gain of mesenchymal markers [21]. It is generally known that EMT-inducing transcription factors Snail, Zeb, and Twist are involved in the transcriptional control and regulatory network that drives tumor progression [22]. However, molecular subtype specific malignant and metastatic cancer cell progression accompanying EMT mechanism remain unclear.

Here we identify SYT11 as a highly expressed gene in stem-like molecular subtype and histologically diffuse-type GC tissues. Moreover, we show that SYT11 plays an important role in GC cell proliferation and metastasis by functioning as scaffold to organize the JNK-MKK7 interactions thereby JNK phosphorylation regulation and EMT-related gene expression, and suggest that SYT11 is a novel prognostic biomarker and therapeutic target against stem-like molecular subtype diffuse GC.

## Materials and methods

### Cell culture

Human GC cell lines AGS, Hs746T, KATOIII, PSK4, MKN-1, MKN-28, MKN-74, NCI-N87, SNU1, SNU16, SNU216, SNU484, SNU601, SNU638, SNU668, and SNU719 were cultured in RPMI-1640 containing 10% fetal bovine serum (FBS). Cell lines YCC2, YCC3, YCC6, YCC7, YCC9, YCC11, and YCC16, generated at Yonsei University College of Medicine, were cultured in MEM containing 10% FBS. HEK293T cells were cultured in DMEM containing 10% FBS. SNU484 and NCI-N87 cells that constitutively expressed SYT11 were selected with 50 μg/ml hygromycin. All cell lines tested for mycoplasma contamination using cycleave polymerase chain reaction (PCR) mycoplasma detection kit (Takara).

### Reagents

We purchased SP600125 from Sigma-Aldrich (St. Louis, MO). SYT11-antisense oligonucleotide (ASO) and NC-ASO were synthesized by Integrated DNA Technologies (Coralville, IA). The ASO sequences were as follows: SYT11-ASO#7 (5′- mA*mU*A*T*G*A*C*A*G*A*G*A*C*A*C*C*T*mG*mG-3′), SYT11-ASO#8 (5′-mU*mU*G*G*C*A*A*T*G*C*G*C*T*T*T*C*T*mG*mC-3′), and NC-ASO (5′-mC*mC*T*A*C*G*C*C*A*C*C*A*A*T*T*T*C*mG*Mu-3′).

### Plasmids

We purchased pCMV3-HA, pCMV3-HA-SYT11, pCMV3-GFP-SYT11, pCMV3-MYC-JNK1, and pCMV3-MYC-JNK2 from Sino Biological (Wayne, PA). Mutations (T1835 and Y185F) in JNK1 were generated using the EZchange site-direct mutagenesis kit (Enzynomics, South Korea). The deletion mutants of GFP-SYT11 were generated using the EZchange site-direct mutagenesis kit. pBluscript-MKK7 was obtained from the Korean UniGene Information. MKK7 was amplified using PCR, then cloned into the pcDNA3.1-Myc or pcDNA3.1-HA plasmid between the EcoRI/BamHI or the EcoRI/XhoI sites, respectively.

### Patients

We performed a retrospective review of a GC cohort database prospectively maintained at Yonsei University College of Medicine (Seoul, South Korea) to identify all patients with gastric adenocarcinoma who underwent curative D2 gastrectomy between 2000 2010 [23]. We obtained demographic and clinicopathologic information and tumor tissue samples from 527-patients. This study was approved by the institutional review board of Severance Hospital (Seoul, Korea; 2015–3104-001). GC samples microarray data were available at the National Center for Biotechnology Information Database of GEO datasets under the data series accession numbers GSE13861 and GSE84437 [23].

### Lentiviral shRNA library construction

Patients with GC were classified by molecular subtypes based on microarray. We selected genes demonstrating an increase beyond twofold in the stem-like molecular subtype compared to the intestinal molecular subtype. Among them, 118 genes exhibited a raw data average of > 500 in the microarray and *p* < 0.05 in the KM analysis. Finally, arrayed 583-shRNA modules were constructed for 118-genes (MISSION shRNA library; Sigma-Aldrich).

### *In vivo* and *in vitro* shRNA screening

We performed shRNA library infection as described previously [24]. Briefly, the shRNA library was packed into a lentivirus via HEK293T cells using Lipofectamine 2000, then transduced into SNU484 cells with a multiplicity of infection of 0.3 and a fold representation of 500. After 48 h of transduction, SNU484 cells were subjected to puromycin selection for 72 h. Transduced SNU484 cells were preserved as a reference sample. For in vivo screening, 5 × 10^6^ cells were injected into mice (*n* = 15). Tumors were extracted 4 to 6 weeks after injection, when their size was about 400 ± 200 mm^3^. For the in vitro screening, cells were harvested weekly for 6 weeks. The shRNA insert barcodes were amplified from the genomic DNA of tissues and cells using Pfu PCR premix (Bioneer, Daejeon, Korea), then sequenced using the Illumina Hi-Seq 2500 system (Illumina). We analyzed the barcode-seq data using the barcode sequence alignment and statistical analysis (Barcas) developed by Kim SY [25].

### Small interfering RNA (siRNA) -mediated gene knockdown

Gene knockdown was performed by introducing siRNA into the target gene using Lipofectamine 2000 (Invitrogen, Carlsbad, CA) according to the manufacturer’s instructions. The siRNA sequences were, as follows: siScramble 5′-CCUACGCCACCAAUUUCGU (dTdT)-3′, siSYT11#4 5′-CAUCAAAGUGCGGAGAGACAA(dTdT)-3′, siSYT11#5 5′-AUC CUUCCUGACAAACGGCAU(dTdT)-3′, siSYT11#6 5′-CCUGCUAAGCCGAGACAAA (dTdT)-3′, siSYT11#7 5′-CCAGGUGUCUCUGUCAUAU(dTdT)-3′, siSYT11#8 5′-GCA GAAAGCGCAUUGCCAA(dTdT)-3′. siRNAs for siANGPTL2 (23452–1), siTHBS4 (7060–1), siJAM3 (83700–1), siVimentin (7431–1), and siMKK7 (5609–1) were purchased from Bioneer.

### Mouse experiments

All animal experiments were approved by the bioethics committee of the Korea Research Institute of Bioscience and Biotechnology. We performed in vivo xenografts as described previously [23]. We injected lentiviral shSYT11 vector infected SNU484 cells (5 × 10^6^) subcutaneously into 5-week-old female BALB/c nude mice. To test SYT11-ASO antitumor efficacy, we injected MKN1 cells (1 × 10^7^) subcutaneously into 5-week-old female BALB/c nude mice. After 1 week, SYT11-ASO [10 mg/kg in 100 μl phosphate-buffered saline (PBS)] was administered via intraperitoneal injection 5 times a week. For the liver metastasis tail vein injection assay, we injected shControl- or shSYT11-expressing lentivirus-infected SNU484 cells (2 × 10^6^), suspended in 100 μl of PBS, into the tail vein of BALB/c nude mice (4 mice per group). After 16 weeks, we removed and fixed the mouse livers. Tumor metastasis to the liver was assessed with Hematoxylin and eosin (H&E) staining. For metastasis quantitation, random field photos were obtained at a magnification of 40× (three fields per mouse) and analyzed using the NIH ImageJ software (version 1.48).

### Western blot analysis

We lysed the cells with RIPA buffer (Millipore, Billerica, MA) containing a protease inhibitor cocktail (Roche, Basel, Switzerland), then quantified the lysates with a protein assay kit (Bio-Rad, Hercules, CA). We used sodium dodecyl sulfate-polyacrylamide gel electrophoresis to separate the cell lysates. Appropriate antibodies were used for protein identification (Supplementary Table 1).

### Reverse transcription-PCR (RT-PCR) and quantitative real-time PCR (qPCR)

Total RNA was isolated using the TRIzol reagent (Invitrogen, Carlsbad, CA). cDNA was synthesized using the TOPscript™ RT DryMIX (Enzynomics, Daejeon, Korea). RT-PCR was performed using the Dr. Taq MasterMix (Doctor Protein, Daejeon, Korea). qPCR was performed using a SYBR Green master mix kit (Qiagen, Valencia, CA). The following primers were used: RPL13A (5′-CTGGACCGTCTCAAGGTGTT-3′ and 5′-TGGTACTTCCAGCCAACCTC-3′), JAM3 [26] (5′-CTGCTGTTCACAAGGACGAC-3′ and 5′-CAGATGCCCAACGTGATCAG-3′). The SYT11 (P281379), GAPDH (P267613), ANGPTL2 (P302397), THBS4 (P266439), and Vimentin (P324997) primers were purchased from Bioneer.

### Live-cell assay for cell proliferation and apoptosis

Cell confluence-based proliferation rates were measured with live-cell imaging (IncuCyte ZOOM System, Essen BioScience, Ann Arbor, MI) as described previously [27]. To analyze apoptosis, we performed kinetic caspase-3/7 measurements using the CellPlayer reagent (Essen BioScience) as described previously [24]. We imaged the cell frames incubated in 96-well plates at 2 h intervals from four separate regions per well using a 10× objective lens. The cultures were maintained in a 37 °C incubator.

### Sulforhodamine B (SRB) and invasion assays

We measured cell viability using the SRB assay as previously described [23]. The cells were fixed with 10% formalin and stained with 0.4% SRB. After 10 min, the cells were washed with 0.01 M acetic acid. The protein-bound dye was dissolved in 10 mM Tris, and its optical density was measured using a spectrophotometer at 540 nm.

For the invasion assays, we used chambers with 8.0-μm-pore PET membrane in 24-well cell culture inserts (BD Biosciences, San Jose, CA). We seeded the cells into the upper part of each chamber with Matrigel coating, whereas we filled the lower compartments with the above-mentioned medium. We then allowed the cells to invade, subsequently fixed them with 10% formalin, and stained with 0.4% SRB.

### Fluorescein isothiocyanate (FITC)-Annexin V/ propidium iodid (PI) double-staining

We performed the FITC-Annexin V/PI double-staining analysis according to the manufacturer’s protocol (BD Biosciences). We treated the cells with ASO, then washed twice with pre-chilled PBS. We stained the treated cells with FITC-Annexin V staining buffer and PI solution for 15 min, then analyzed with a FACSCalibur Flow Cytometer (BD Biosciences).

### Immunohistochemistry (IHC)

Tissue array blocks of human GC and normal tissues were supplied by US Biomax (Rockville, MD). IHC was performed as previously described [23]. We incubated the slides with anti-SYT11 antibodies. After washing with PBS, we incubated the slides with biotinylated anti-rabbit IgG (Vector Laboratories, Burlingame, CA) and avidin-biotin-peroxidase (Vector Laboratories), then visualized using diaminobenzidine tetrahydrochloride (Vector Laboratories). We counterstained the sections with hematoxylin.

### Immunofluorescence staining

Immunofluorescence staining was performed as described previously [27]. Cells were incubated with anti-SYT11, anti-MKK7, and anti-JNK antibody at 4 °C O/N and then incubated with secondary fluorescent (Alexa-546 or FITC) antibodies for 1 h. Subsequently, the cells were counterstained with 4′,6-diamidino-2-phenylindole (DAPI) and analyzed under a confocal microscope (LSM 5 LIVE DuoScan, Carl Zeiss, Stuttgart, Germany).

### Spheroid formation

We induced three-dimensional spheroid cultures as described previously [23, 28].

### Statistical analyses

To compare the gene expression differences between the patient subgroups, we performed two-sample t-tests. We calculated Pearson’s correlation coefficients to evaluate genetic associations. To divide the patients into two single gene expression subgroup, we obtained an optimal gene expression cutoff from the ROC analysis, determining the best cutoff by the expression with the highest multiply of sensitivity and specificity. Statistical analyses were performed using MedCalc version 18.11.6 (MedCalc Software, Ostend, Belgium). We used the Kaplan-Meier method to calculate the time before death and measured the difference between the times using the log-rank test.

## Results

### Genome-wide screening of GC tissues revealed a high SYT11 expression in the stem-like molecular subtype

**Fig. 1:**
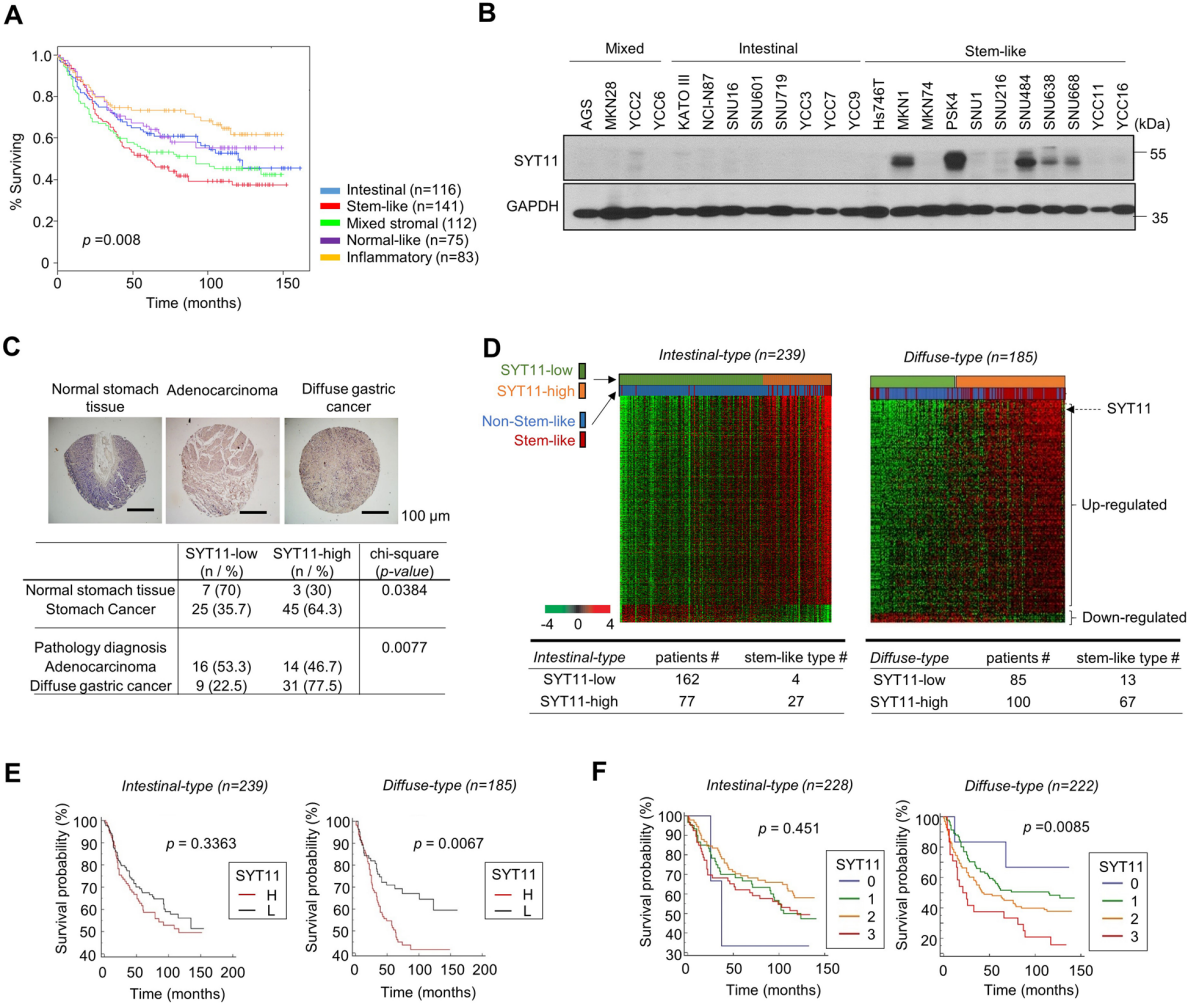
Effect of synaptotagmin 11 (SYT11) on the survival rate of diffuse-type GC patients. **A,** The overall survival (OS) of GC patients with each molecular subtype of the YSH (Yonsei University Severance Hospital) cohort. **B,** SYT11 protein expression level was compared between various GC cells with western blotting. **C,** Immunohistochemistry (IHC) analysis of normal and GC tissues (US Biomax). Statistical significance was performed with the χ^2^ test. **D,** Heatmap of gene expression in patients with intestinal-type (*n* = 239) or diffuse-type (*n* = 185) GC (YSH cohort). **E,** Kaplan-Meier curves of the OS of intestinal-type (*n* = 239) and diffuse-type (*n* = 185) GC patients stratified by SYT11 mRNA expression in a microarray. **F,** Kaplan-Meier survival for SYT11 protein expression by IHC analysis of GC patient tissues (YSH cohort, intestinal-type *n* = 228 and diffuse-type *n* = 222). SYT11 expression level was classified from 0 to + 3

From the transcriptome analysis of 527 patients with GC (Yonsei University Severance Hospital, YSH, Seoul, Korea), we identified five molecular subtypes: intestinal, mixed stromal, normal-like, inflammatory, and stem-like [3], the latter exhibiting unfavorable survival rate (Fig. 1A). Our gene ontology analysis revealed that vascular smooth muscle contraction-, focal adhesion-, and tight junction-related genes were up-regulated in the stem-like molecular subtype samples (Supplementary Fig. 1A and B).

To identify potential stem-like molecular subtype GC therapeutic targets, we selected the 118 genes that were more highly expressed in the stem-like molecular subtype than the intestinal molecular subtype. After short hairpin RNA (shRNA) library preparation, we performed RNA interference screening both in vitro and in vivo in SNU484 cells, which are stem-like subtype cells, as described in Supplementary Fig. 1C. We then analyzed barcodes/shRNAs using the barcode sequence alignment and statistical analysis (BARCAS) [25] and selected 30 genes related to reduced viability both in vitro and in vivo*.* Then, we examined SNU484 cells growth inhibition upon the treatment with the shRNA of the corresponding genes. We selected SYT11 as the most efficient gene for SNU484 cell viability inhibition (Supplementary Fig. 1D).

Next, we compared the previously reported SYT family gene expression in GC with that of the patient samples in microarray analysis. The SYT11, but not the SYT7, SYT8 and SYT13, mRNA expression elevated in stem-like molecular subtype compared with the intestinal molecular subtype patient samples (Supplementary Fig. 2A). In addition, we analyzed the SYT11 mRNA expression in the different molecular subtypes of GC cell lines, and observed high-level SYT11 protein expression in stem-like molecular subtype GC cell lines (Fig. 1B). These results suggest that SYT11 is selectively expressed in stem-like molecular subtype GC.

### SYT11 expression can stratify patients with GC

In order to investigate the function of SYT11 expression in GC, the SYT11 protein expression level was examined in different gastric tissues types (*n* = 80) via IHC analysis. GC tissues exhibited a high-level SYT11 protein expression (64.3%) compared with normal gastric tissue (30%) (Fig. 1C). We observed that 77.5 and 46.7% of the diffuse-type GC tissue and adenocarcinoma samples exhibited a high-level SYT11 protein expression, respectively (Fig. 1C). We then compared SYT11 mRNA levels between intestinal- and diffuse-type GC tissues according to the Lauren classification. Our GC tissue microarray analysis showed that the number of the patient tissues with high SYT11 expression was higher in the diffuse-type disease than that in the intestinal-type (Fig. 1D). We also investigated how SYT11 mRNA affects patient survival. High SYT11 mRNA expression in diffuse-type tumors significantly affected patient survival but not in patients with the intestinal-type disease (Fig. 1E). We then examined whether the SYT11 protein level affects survival performing IHC on the tissue assay samples (Fig. 1F). As expected, higher SYT11 protein expression was associated with worse survival in diffuse-type GC (Fig. 1F). The SYT11 expression level significantly affected patient survival, particularly in the diffuse-type cancer setting. This result indicates that SYT11 expression could be a potential prognostic marker for predicting patient survival.

### SYT11 induces JNK1 phosphorylation by JNK-MKK7 complex formation

**Fig. 2:**
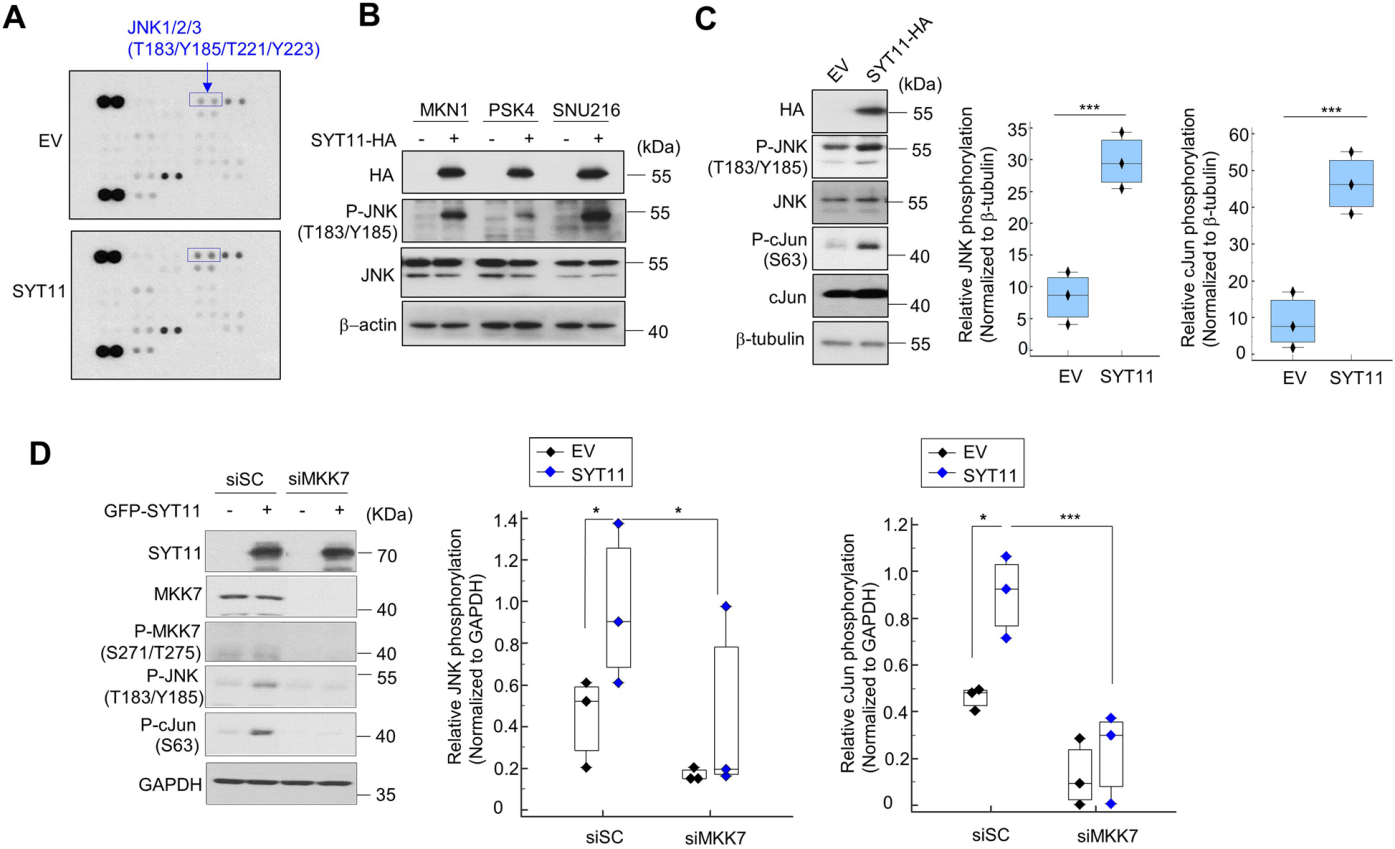
Phosphorylation of JNK induced by SYT11 overexpression. **A,** Proteome profile phospho-kinase array (R&D Systems) of SYT11-overexpressing SNU484 (SNU484-SYT11) cells. Stable SNU484 cells were generated by transfection of the *SYT11* gene containing vector (pCMV3-HA-SYT11) or the control vector (empty vector, pCMV3-HA). **B,** Induction of the JNK phosphorylation in various GC cells (MKN1, PSK4 and SNU216) overexpressing SYT11. Cells were transfected with pCMV3-HA or pCMV3-HA-SYT11. SYT11 expression was analyzed by western blot. **C,** Increased phosphorylation of JNK and cJun by SYT11. JNK and cJun phosphorylation in SNU484-SYT11 and SNU484-EV cells was analyzed by western blot with the corresponding antibodies. The density of each band was analyzed using the ImageJ software (*n* = 3). **D,** Inhibition of SYT11-induced JNK and cJun phosphorylation in the absence of MKK7 expression (siMKK7). The level of phosphorylation of JNK and cJun was analyzed using the ImageJ software (*n* = 3)

Next, we performed phospho-kinase array to investigate whether SYT11-related intracellular signaling is involved. Compared with empty vector (EV)-expressing SNU484 cells, JNK phosphorylation increased in SYT11-overexpressing SNU484 cells (Fig. 2A) and MKN1, PSK4, and SNU216 cells transiently transfected with SYT11 (Fig. 2B). High-level SYT11 expression also increased cJun phosphorylation (Fig. 2C). However, it did not affect the MKK7 phosphorylation, which is upstream of JNK (Fig. 2D). Interestingly, neither SYT11-related JNK nor cJun phosphorylation was observed in MKK7 knocked-down cells (Fig. 2D), indicating that MKK7 is required for the SYT11-related JNK and cJun phosphorylation.

**Fig. 3:**
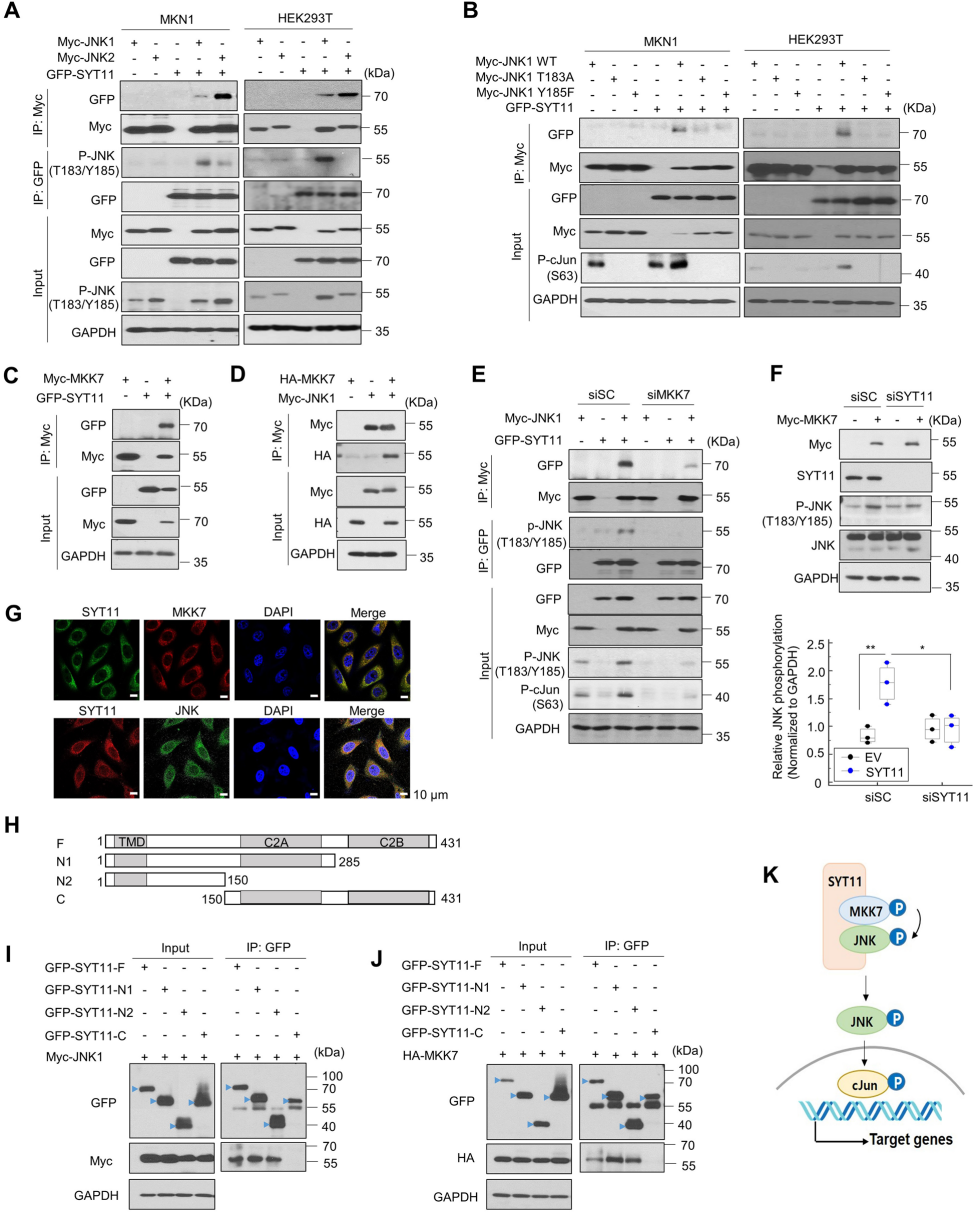
SYT11 stabilizes JNK phosphorylation via SYT11-MKK7-JNK complex formation. **A,** The interaction of SYT11 with phosphorylated JNK1: MKN1 and HEK293T cells were transfected with Myc-JNK1 or Myc-JNK2 alone or together with GFP-SYT11. Cell lysates were used for an immunoprecipitation assay using an anti-Myc or anti-GFP antibody. **B,** Binding of SYT11 and mutant JNK1: MKN1 and HEK293T cells were transfected with Myc-JNK1 WT, Myc-JNK1 T183A, or Myc-JNK1 Y185F alone or together with GFP-SYT11. Cell lysates were used for an immunoprecipitation assay using anti-Myc antibody. **C,** SYT11 interaction with MKK7: MKN1 cells were transfected with Myc-MKK7 and GFP-SYT11. Cell lysates were used for an immunoprecipitation assay using anti-Myc. **D,** JNK1 interaction with MKK7: HEK293T cells were transfected with Myc-JNK1 and HA-MKK7. **E,** Requirement of MKK7 for interaction between SYT11 and JNK1. **F,** Requirement of SYT11 for cJun phosphorylation by MKK7. The level of phosphorylation of JNK and cJun was analyzed using the ImageJ software (*n* = 3). **p ≤ 0.01; *p ≤ 0.05 (Student’s t-test). **G,** Co-localization of SYT11 with JNK or MKK7 in MKN1 cells. **H,** Deletion mutants of GFP-SYT11. **I** and **J**, HEK293T cells were transfected with the deletion mutants of GFP-SYT11, Myc-JNK1, and HA-MKK7 in the indicated combinations. Cell lysates were immunoprecipitated using anti-GFP antibody. **K,** Proposed JNK phosphorylation by SYT11 and MKK7

To understand the mechanism behind SYT11-mediated JNK phosphorylation, we performed an immunoprecipitation assay using GFP-SYT11-expressing cells. The SYT11-JNK1 or SYT11-JNK2 interaction occurred in JNK1-SYT11 or JNK2-SYT11 co-transfected MKN1 and HEK293T cells (Fig. 3A). SYT11 exhibited a particularly strong interaction with phosphorylated JNK1 (Fig. 3A). To validate whether JNK phosphorylation was required for SYT11 binding, we mutated the T183 and Y185 phosphorylation sites in JNK1. In MKN1 and HEK293T cells containing these mutation, JNK1 did not bind SYT11, resulting in no cJun phosphorylation (Fig. 3B). Moreover, our immunoprecipitation assay confirmed the direct binding of SYT11 and MKK7 (Fig. 3C), which is required for JNK phosphorylation. MKK7-JNK1 binding could also be observed (Fig. 3D). As expected, the JNK1-SYT11 binding was inhibited in the MKK7 knocked-down cells (Fig. 3E), suggesting that MKK7 is required for the phosphorylation of JNK through their binding. Moreover, MKK7-induced JNK phosphorylation did not occur in SYT11- knocked-down SNU484 cells (Fig. 3F).

Then, immunofluorescence staining was performed to confirm SYT11 and JNK or MKK7 co-localization in cells. The fluorescence expression of SYT11–JNK and SYT11–MKK7 in MKN1 cells was confirmed to be colocalized in the cytoplasm or Golgi region (Fig. 3G). Furthermore, we constructed SYT11 deletion mutants to identify which region of SYT11 binds to JNK or MKK7 (Fig. 3H). By immunoprecipitation assay, JNK1 and MKK7 were found to interact with the SYT11 N-terminus containing the transmembrane domain but not with the C-terminus containing the C2A and C2B domains (Fig. 3I and J). This suggests that SYT11 forms a complex with MKK7 and JNK1, leading to JNK induction and stabilization as well as cJun phosphorylation to permit further transcriptional regulation (Fig. 3K).

### SYT11 is involved in EMT-related gene regulation

**Fig. 4:**
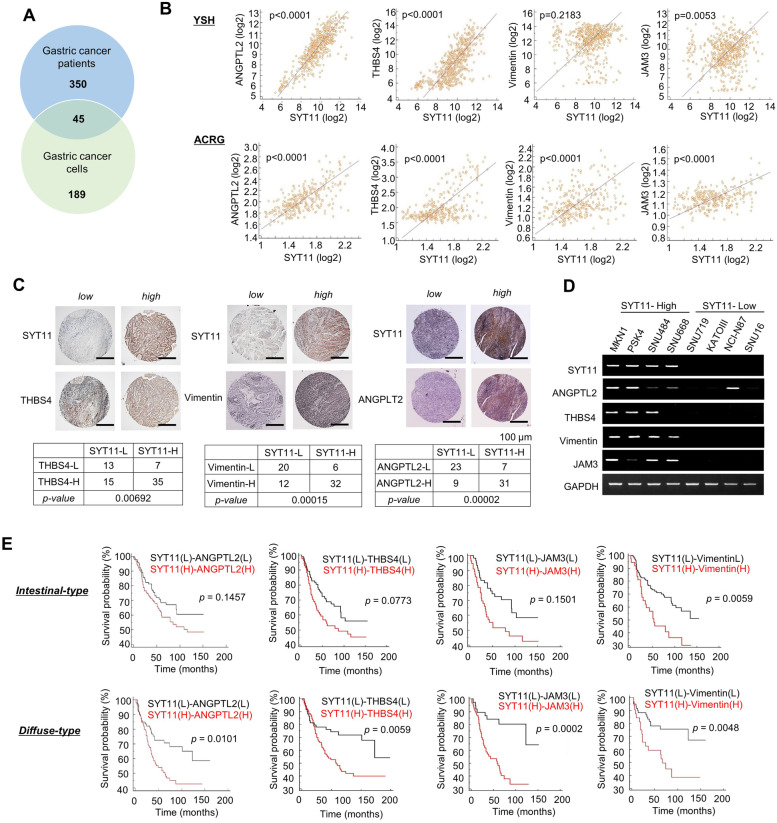
SYT11-associated genes. **A,** Venn diagrams of up-regulated genes in GC patient tissues or GC cells expressing high levels of SYT11 (fold change > 1.5; *p* < 0.001). **B,** Genes that are highly correlated with SYT11. The expression correlation of SYT11 with THBS4, JAM3, Vimentin and ANGPTL2 was examined in the YSH cohort (*n* = 527) and ACRG cohort (GSE66229; *n* = 300). **C,** IHC analysis of THBS4, Vimentin, ANGPTL2 and SYT11 in GC tissues (US Biomax). More THBS4, Vimentin and ANGPTL2 were present in SYT11-high tissues. **D,** mRNA level of the indicated genes in SYT11-high or SYT11-low GC cell lines. **E,** Kaplan-Meier survival curves comparing high or low SYT11 expression and the genes ANGPTL2, THBS4, JAM3 and Vimentin in intestinal-type and diffuse-type GC

To identify the genes associated with SYT11 function, we investigated a group of genes in SYT11-high and SYT11-low expressing tissue samples of patients with GC or GC cell lines via transcriptome analysis. Out of 350 and 189 genes associated with high SYT11 expression in tissue samples of patients with GC and GC cell lines, respectively, we selected 45 genes with an increased expression both in the tissue samples and cell lines (Fig. 4A, Supplementary Table 2). We then correlated these genes with SYT11 expression in the YSH and ACRG cohort and selected angiopoietin-like 2 (ANGPTL2), thrombospondin 4 (THBS4), Vimentin, and junctional adhesion molecule 3 (JAM3) as highly co-expressing genes with SYT11 (Fig. 4B). The IHC analysis of patients tissue samples with GC also showed a correlation between the high SYT11 expression and that of THBS4, Vimentin, and ANGPTL2 (Fig. 4C). ANGPTL2, THBS4, Vimentin, and JAM3 mRNA expression also increased in GC cell lines with high-level SYT11 expression (Fig. 4D). Furthermore, higher SYT11-ANGPTL2, SYT11-JAM3 and SYT11-THBS4 expression indicated poorer prognosis, especially in patients with diffuse-type GC compared to those with intestinal-type disease (Fig. 4E).

**Fig. 5:**
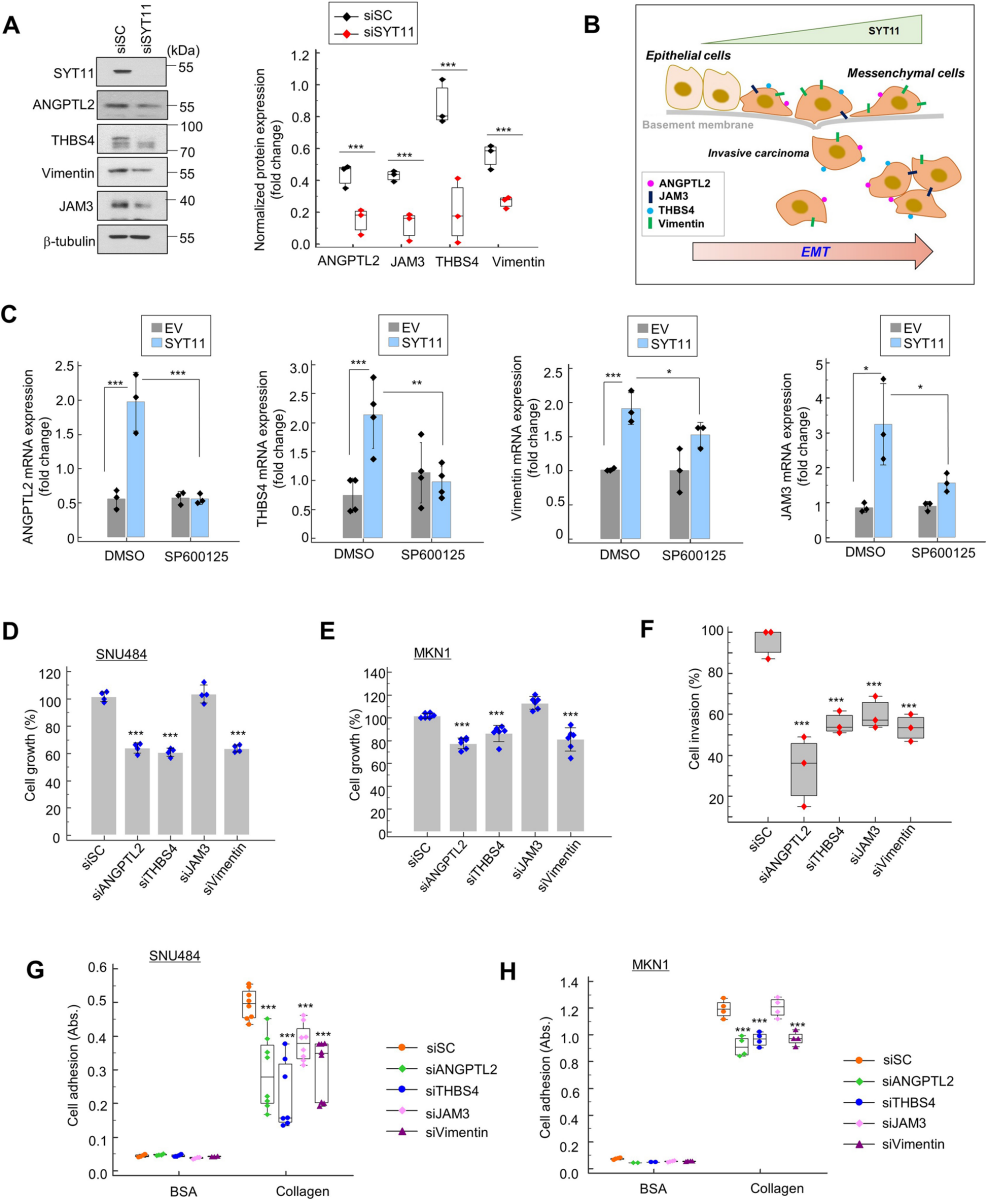
JNK is involved in the SYT11-induced expression of EMT genes. **A,** Protein levels of the EMT genes in SNU484 cells transfected with siSYT11#8 or siScramble were analyzed by western blotting. The density of each band was analyzed using the ImageJ software (*n* = 3). **B,** EMT induction in cancer cells according to the increase of SYT11 expression level. **C,** Inhibition of mRNA expression of ANGPTL2, THBS4, JAM3, and Vimentin by the JNK inhibitor, SP600125, in the presence of high SYT11-expression in MKN1 cells as shown by qPCR. *n* = 3. ***p ≤ 0.005; **p ≤ 0.01; *p ≤ 0.05 (Student’s t-test). **D** and **E,** SNU484 and MKN1 cells were treated with 40 nM each of siRNA for 48 h. Cell viability was analyzed with the SRB assay (*n* = 6). ***p ≤ 0.005 (Student’s t-test). **F,** Invasion activity of SNU484 cells transfected with siScramble, siANGPTL2, siTHBS4, siJAM3, or siVimentin for 48 h (*n* = 3). **G** and **H,** Adhesion assay of SNU484 and MKN1 cells transfected with siScramble, siANGPTL2, siTHBS4, siJAM3 or siVimentin. Cells were treated with 40 nM each of siRNAs for 48 h. After trypsin treatment, the suspended cells were placed in a collagen-coated 96-well plate. After 1 hr., cells that did not adhere to the floor were washed off with PBS. After staining with SRB, the absorbance of cells bound to collagen was measured (*n* = 6)

To further investigate the functional correlation between the selected EMT associated genes and SYT11, we prepared siRNAs for SYT11. Of the siRNAs used, siSYT11#8 inhibited most SYT11 expression and SNU484 cell growth (Supplementary Fig. 3A and B). SYT11 knockdown by siSYT11#8 decreased the ANGPTL2, THBS4, Vimentin, and JAM3 protein expression levels (Fig. 5A), suggesting that SYT11 regulates the expression of their encoding genes. To investigate whether the SYT11-induced JNK phosphorylation could be involved in other downstream expression, we examined ANGPTL2, THBS4, Vimentin, and JAM3 mRNA expression in SYT11-transfected MKN1 cells. SYT11 overexpressing MKN1 cells displayed a high expression of these genes (Fig. 5C). However, when these cells were treated with the JNK inhibitor SP600125, the mRNA expression of these genes was suppressed even in the presence of high-level SYT11 (Fig. 5C). These results suggest that the SYT11-mediated induction of ANGPTL2, THBS4, Vimentin, and JAM3 gene expression requires JNK phosphorylation. As these genes are involved in cell adhesion and EMT induction, we evaluated their SYT11 expression-related influence on GC cell proliferation, invasion, and adhesion. Notably, ANGPTL2, THBS4, Vimentin, and JAM3 knockdown significantly inhibited high-level SYT11-expression SNU484 and MKN1 cell proliferation, invasion, and adhesion (Fig. 5D-H). These results suggest that SYT11 induces EMT by regulating cell adhesion- and invasion-related genes (Fig. 5B).

### SYT11 regulates GC proliferation and metastasis

**Fig. 6:**
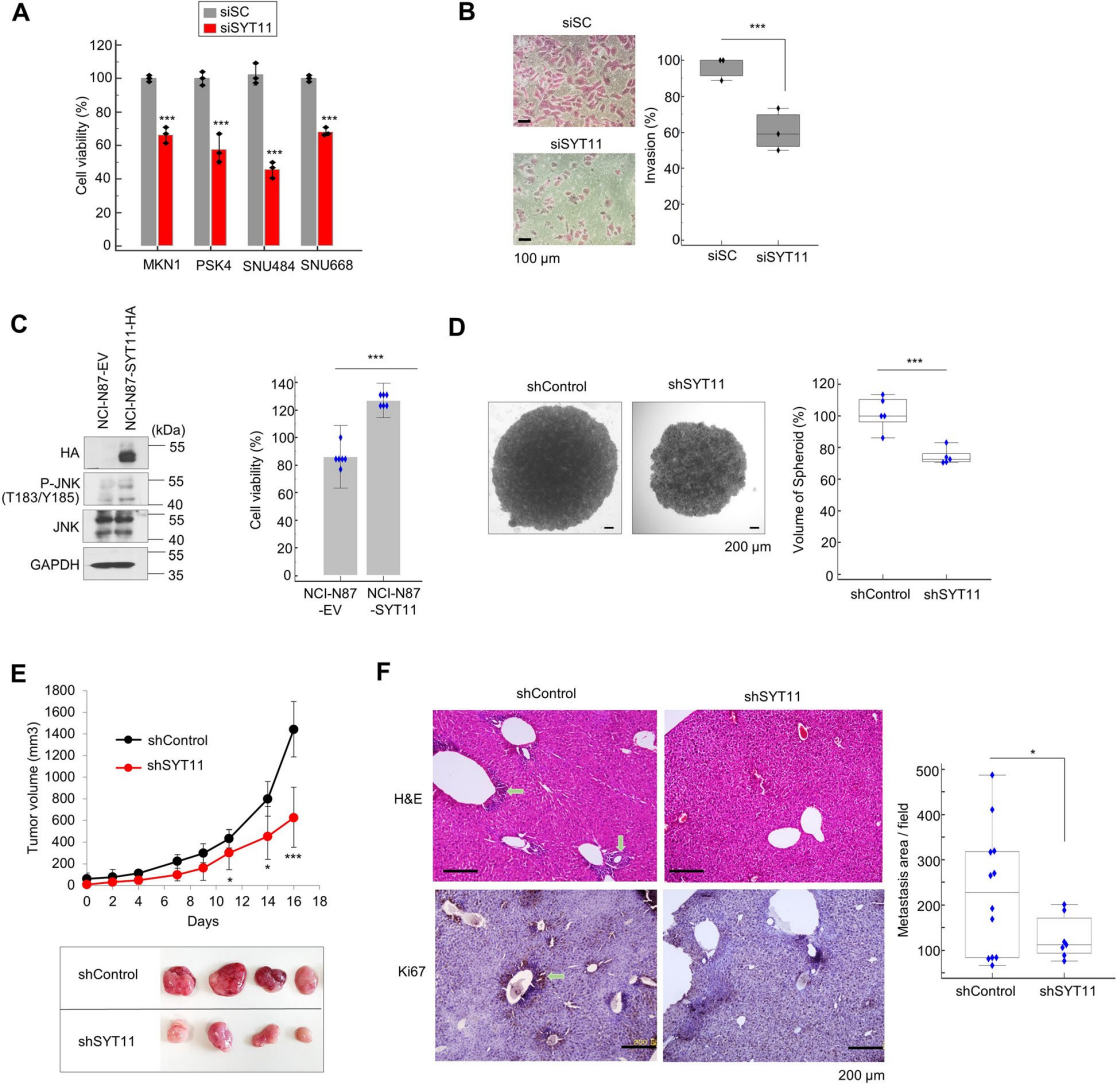
SYT11 is involved in GC cell proliferation and metastasis. **A,** In vitro growth-inhibitory effects of SYT11 knockdown in GC cells. GC cells expressing high levels of SYT11 were treated with 40 nM siSYT11 or siScramble for 48 h, and cell viability was analyzed with the SRB assay (*n* = 3). **B,** In vitro invasion assay of SNU484 cells transfected with 40 nM siSYT11 or siScramble (*n* = 3). **C,** Increase in the cell growth in NCI-N87 cells with low levels of SYT11 expression via inducing SYT11 overexpression: The expression of proteins was analyzed via western blotting. Cell viability was analyzed using SRB assay (*n* = 6). **D,** Inhibition of Spheroid formation of SNU484 cells treated with lenti-shSYT11#5 (*n* = 5). Scale bar, 200 μm. **E,** Inhibition of tumor formation by SYT11 knockdown: In vivo tumor formation was performed using SNU484 cells infected with lenti-shSYT11#5 or lenti-shControl. **F,** Inhibition of metastasis to the liver by shSYT11. Tail vein injection assay of SNU484 cells expressing shSYT11#5. H&E and IHC staining for Ki-67 in the liver 16 weeks after injection. Scale bar, 200 μm. ***p ≤ 0.005; **p ≤ 0.01; *p ≤ 0.05 (Student’s t-test)

Next, we examined how SYT11 could affect GC cell growth and invasion. First, we observed that siSYT11-treated GC cell lines with intrinsically high SYT11 expression inhibited cell growth (Fig. 6A) and invasion (Fig. 6B). We also found that SYT11-overexpressing stable cells (NCI-N87-SYT11-HA) generated from NCI-N87 cells minimally expressing SYT11 exhibited increased cell proliferation compared with control cells (NCI-N87-EV) (Fig. 6C). shSYT11-expressing lentivirus-infected SNU484 cells exhibited reduced spheroid formation compared with the control (Fig. 6D). SYT11 affected similarly tumor formation in in vivo xenografts using shSYT11-expressing lentivirus-infected SNU484 cells (Fig. 6E). shSYT11- or shControl-expressing lentivirus-infected SNU484 cells were injected into the mouse tail vein to investigate how shSYT11 affects hematogeneous metastasis. In H&E or anti-Ki67 antibody-stained liver tissues after 16 weeks, GC metastasis to the liver was suppressed in mice injected with shSYT11-expressing SNU484 cells compared with the control (Fig. 6F). Furthermore, we produced SYT11 transgenic (SYT11-Tg) mice to confirm the effect of SYT11 overexpression on tumor metastasis. The human SYT11 mRNA expression increased in the liver, lung and stomach of SYT11-Tg mice (Supplementary Fig. 4A). After 18 days of transplanting the mouse melanoma B16F10-Luc cells into the mouse tail vein, increased lung metastasis was observed in SYT11-Tg mice compared with WT mice (Supplementary Fig. 4B and C). These results suggest that SYT11 plays an important role in GC cell proliferation and metastasis.

### SYT11-ASO exhibits an antitumor effect on GC cells

**Fig. 7:**
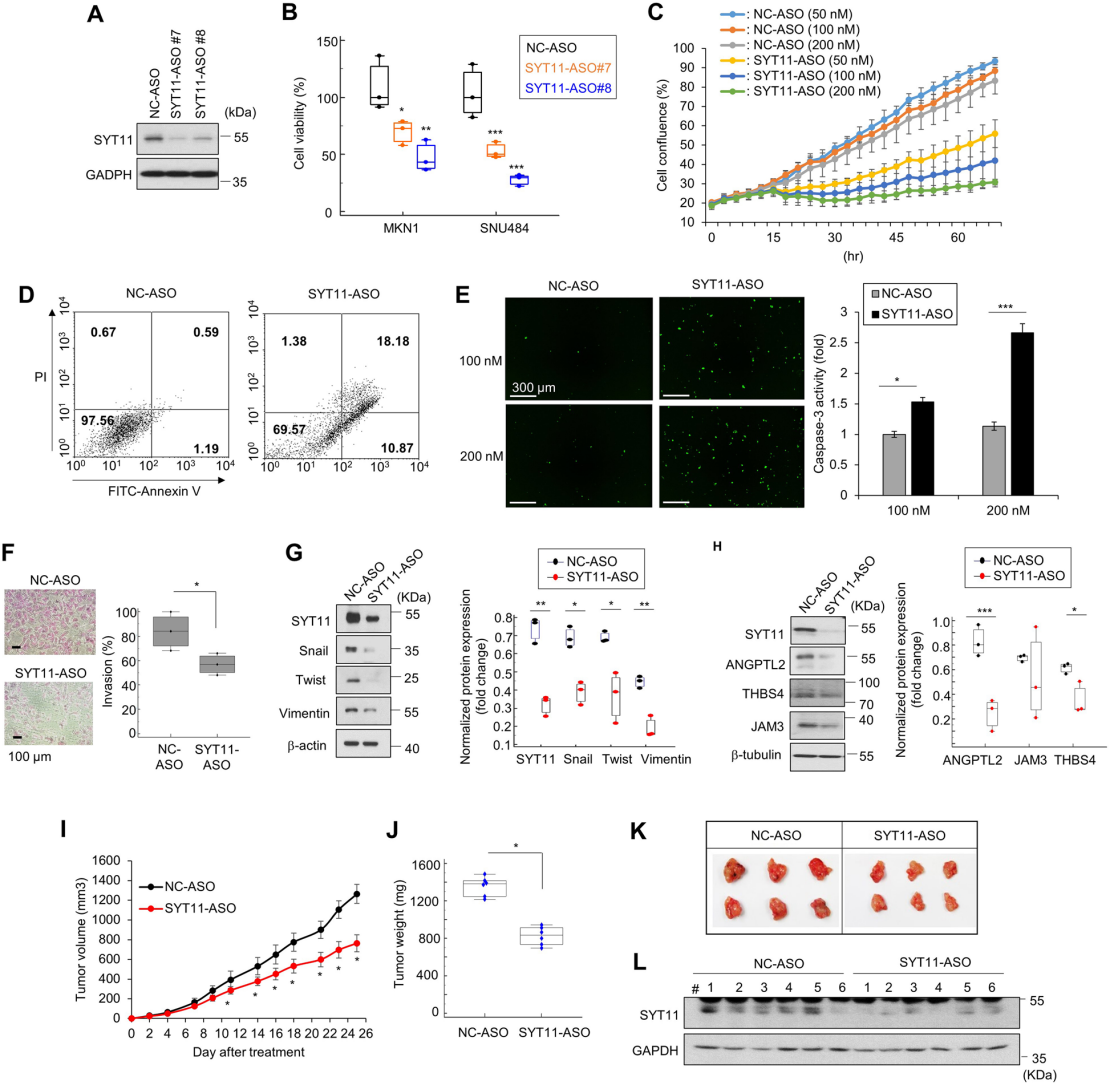
Antitumor effects of SYT11-ASO. **A,** Inhibition of SYT11 expression with an antisense oligonucleotide (ASO) of SYT11. SNU484 cells were treated with 50 nM SYT11-ASOs for 48 h. SYT11 expression was analyzed by western blot. **B,** Inhibition of cell viability by SYT11-ASO. SNU484 and MKN1 cells were treated with 50 nM SYT11-ASOs for 48 h. Cell viability was analyzed by the SRB assay (*n* = 3). **C,** Growth inhibition of SNU484 cells treated with various concentrations of SYT11-ASO #8 or NC-ASO. Cell growth was analyzed with the live-cell imaging system (*n* = 12). **D,** Scatter grams of FITC-Annexin V/PI staining of SNU484 cells treated with 100 nM SYT11-ASO #8 and NC-ASO. **E,** Caspase-3 activity of MKN1 cells treated with SYT11-ASO #8 and NC-ASO (*n* = 12). **F,** Inhibition of the invasion of SNU484 cells treated with SYT11-ASO#8 (*n* = 3). **G,** Inhibition of the expression of EMT-related proteins in SNU484 cells treated with SYT11-ASO #8 as analyzed by western blotting (*n* = 3). **H,** Reduced expression of genes downstream of SYT11 using SYT11-ASO #8 in SNU484 cells. **I-K,** The antitumor effects of SYT11-ASO in an in vivo xenograft model. SYT11-ASO#8 (10 mg/kg) was administered intraperitoneally in the MKN1 mouse model 5 times a week (*n* = 6). **L.** SYT11 expression in tumor tissues analyzed by western blot. ***p ≤ 0.005; **p ≤ 0.01; *p ≤ 0.05 (Student’s t-test)

To develop a SYT11-mediated signaling-based anticancer agent against GC, we designed and synthesized SYT11-ASO. Reduced SYT11 expression in SYT11-ASO#7 or #8-treated cells resulted in substantial growth inhibition of MKN1 and SNU484 (Fig. 7A and B). In a real-time cell analysis system, cell growth was reduced in a concentration-dependent manner for SYT11-ASO (Fig. 7C). Furthermore, our Annexin V- and PI-staining-based fluorescence-active cell sorting analysis showed that SYT11-ASO induced apoptosis in SNU484 cells (Fig. 7D). The caspase-3 activity showed an increase in SYT11-ASO-treated cells compared with the negative control (NC)-ASO-treated cells (Fig. 7E). SYT11-ASO also inhibited SNU484 cell invasion (Fig. 7F). SYT11-ASO inhibited the expression of the EMT-related genes Snail, Twist, and Vimentin (Fig. 7G). In addition, the SYT11 downstream ANGPTL2, THBS4, and JAM3 gene expression were also reduced upon the SYT11-ASO treatments (Fig. 7H). We then observed that SYT11-ASO reduced tumor formation in a xenograft model (Fig. 7I-L). These results suggest that SYT11-ASO exhibits an antitumor effect by inducing GC cell apoptosis and inhibiting EMT.

## Discussion

The molecular subtyping of malignancies is important for proper cancer diagnosis and therapeutic selection. Genome-scale analyses of colorectal [29], breast [30], and gastric [1, 2] cancers have identified potential biomarkers for clinical diagnosis, prognosis, and therapeutic targets. Synaptotagmin 11 (SYT11) has been previously known as a critical mediator of parkin-linked neurotoxicity [31]. The present study identified SYT11 as a potential prognostic GC marker, especially for the stem-like molecular subtype, following the transcriptome analysis of 527 tissue samples of patients with GC and verification using GC cell lines. SYT11 is frequently expressed in patients with histologically classified diffuse-type GC, where high SYT11 expression is associated with a poor prognosis. Moreover, SYT11 knockdown also inhibited the growth and invasion of stem-like molecular subtype GC cell lines. Furthermore, the SYT11-ASO treatment also similarly affected GC cells and in a mouse model, confirming that SYT11 can be a potential novel GC therapeutic target.

Until now, no study described any GC progression-related effector mechanism of SYT11. The present study revealed that SYT11 is involved in the MKK7-JNK phosphorylation cascade in GC development. JNK reportedly either promotes or inhibits carcinogenesis depending on the cells of origin [32, 33]. As gastric carcinogenesis is induced by NMU, JNK1 deficiency reduced tumor formation [34]. JNK also influences chemotherapy resistance through autophagy induction, crosstalk with other signaling pathways such as nuclear factor-κB, p38, and JunD, and compensatory cell proliferation activation [32]. We found that JNK expression was high in stem-like molecular subtype GC cells and that JNK knockdown inhibited GC cell growth (Supplementary Fig. 5). We also observed that SYT11 binds only to the phosphorylated form of the JNK-MKK7 complex. Scaffold proteins such as JIP, JLP, POSH, and WDR62 form an assembly with the JNK module to activate JNK-mediated cellular responses [35–37]. In this case, each kinase protein was individually bound to the docking sites of the scaffold protein. JIP1 typically formed complexes with MLK, MKK4/7 and JNK, phosphorylated JNK, and translocated to the nucleus. Meanwhile, WDR62 formed complexes with MKK7 and JNK and phosphorylated JNK but did not affect cJun phosphorylation [36]. Another scaffold protein, MEKK1, is the protein kinase that combines with another protein kinase to form a signaling module. Based on this study, SYT11 likely functions as a scaffold protein that forms a complex with MKK7 and JNK to stimulate JNK phosphorylation for the MKK7-JNK-cJun cascade signaling.

Gene ontology analysis data showed that patients with stem-like molecular subtype GC exhibited the up-regulation of focal adhesion-, vascular smooth muscle contraction-, and tight junction-related genes compared to those with intestinal molecular subtype GC, suggesting that these are direct downstream SYT11 targets. ANGPTL2 is known to promote cell proliferation, metastasis, and EMT in lung cancer, breast cancer, hepatocellular carcinoma, and pancreatic ductal adenocarcinoma [38–41]. ANGPTL2 is a reported prognostic and diagnostic marker of GC and colorectal cancer [42, 43]. Vimentin is also regulated by cJun, c-Fos and Smad3 through the AP-1 binding site [44]. In particular, Vimentin is a known EMT marker, and our SYT11 knockdown confirmed the SYT11-mediated expression regulation of Snail and Twist, as well as Vimentin, suggesting that SYT11 plays a crucial role in the control of EMT gene expressions. THBS4 was identified as a diffuse-type gastric adenocarcinoma marker [45], and described to promote GC cell proliferation and metastasis [46]. Interestingly, THBS4 and ANGPTL2 are highly expressed in patients with stem-like molecular subtype GC (Supplementary Fig. 2B), these two genes might thus be used as companion diagnostic markers for SYT11 therapy. JAM3, an adhesion and transmigration regulatory element, has been identified as a tumor suppressor gene through DNA methylation in colorectal cancer [26], but it has also been described to inhibit apoptosis and induce cell migration in renal carcinoma [47]. In GC, there is only one study reporting higher JAM3 expression in cancer tissues compared to healthy controls [48], and no studies on its related functions or effector mechanisms has been published yet. In this study, we described that JAM3 expression decreased in SYT11-knocked-down cells. JAM3 inhibition led to reduced cell invasion and adhesion. In addition, since SYT11-mediated JNK phosphorylation can regulate downstream gene expression, we observed that SYT11expression highly correlated with that of ANGPTL2, THBS4, JAM3, and Vimentin, prognostic biomarkers of diffuse-type GC.

Other than HER2-trageted drugs, no targeted therapeutics are currently available for GC treatment, especially for diffuse-type disease. In this study we discovered SYT11 as a prognostic marker and validated it as a novel therapeutic target of diffuse-type GC. The elucidation of SYT11 function as a scaffold protein for MKK7-JNK phosphorylation could be an excellent step toward the development of novel therapeutic agents for GC. Seven RNA-targeted therapeutic drugs have been approved by the US Food and Drug Administration since Vitravene in 1998. RNA-targeting therapeutic technologies include mRNA, antisense RNA, miRNA, siRNA, and RNA aptamers. ASO has advantages over these due to its easier administration, relatively easy intracellular delivery, and easy applicability in various tissues. The ASO against SYT11 was designed and synthesized as an anticancer agent based on SYT11-mediated signaling. SYT11-ASO showed growth inhibition, apoptosis induction, and metastasis suppression in both in vitro and in vivo mouse models. As combination therapy is preferred against most cancers, finding a combination therapy drug with SYT11 is essential for increasing treatment efficacy.

## Conclusions

In summary, SYT11 is highly expressed in the stem-like molecular subtype of GC, and could also serve as a prognostic biomarker in diffuse-type GC. SYT11 forms a complex with MKK7-JNK, allowing JNK and cJun phosphorylation. Phosphorylated cJun induces ANGPTL2, THBS4, JAM3, and Vimentin downstream gene expression, leading to GC cell oncogenesis. This study also demonstrates SYT11 as a potential therapeutic target against diffuse-type GC and recommends SYT11-ASO as a potential anticancer agent for diffuse-type GC.

## Supplementary Information

**Additional file 1: Supplementary Figure 1.** Identification of SYT11 as a marker for the stem-like molecular subtype of GC. A and B, Gene Ontology analysis by molecular subtype in GC patients. C, Schematic diagram of in vitro and in vivo RNA interference (RNAi) screening. D, The effect of gene knockdown in stem-like GC cells during the RNAi screen. SNU484 cells were infected with lentiviral shRNAs of each gene for 48 h. Cell viability was analyzed by SRB assay (*n* = 3). **Supplementary Figure 2.** Comparison of gene expression by molecular subtype of patients with GC. A, The mRNA expression of SYT7, SYT8, SYT11, and SYT13 in GC patients with intestinal (*n* = 116) or stem-like (*n* = 141) molecular subtypes compared with a two-tailed t-test. B, The mRNA expression level of THBS4, Vimentin, JAM3, and ANGPTL2 in GC patients with intestinal molecular subtype (*n* = 116) or stem-like molecular subtype (*n* = 141). **Supplementary Figure 3.** SYT11 expression and cell proliferation after SYT11 knockdown. A, SNU484 cells were treated with 20 nM siSYT11 for 48 h. The mRNA expression of SYT11 and RPL13A was measured with RT-PCR. PSK4 cells were treated with 20 nM siSYT11 for 48 h. The protein expression of SYT11 was analyzed with a western blot. B, SNU484 cells were treated with 20 nM siSYT11 for 48 h. Cell viability was analyzed with the SRB assay (*n* = 3). **Supplementary Figure 4.** Effect of SYT11 on lung metastasis in mice. A, SYT11-HA transgenic mice were created by Macrogen (Seoul, Korea). F2 generation male mice were used for the animal study. For human SYT11 genotyping, we used the following primers: F-5′-GTGGATAGCGGTTTGACTCAC-3′ and R-5′-GAAGGTCTCGTCAAACACAGG-3′. Total RNA was extracted from the tissue of WT and SYT11-Tg mice (RNeasy mini kit, Qiagen, Valencia, CA). The mRNA expression of human SYT11 and mouse GAPDH was analyzed with RT-PCR. B, Genotype identification of SYT11 transgenic mouse. Genomic DNA was extracted from the tail of the mouse for genotyping (Wizard genomic DNA purification kit, Promega Madison, WI). In WT and SYT11-Tg mice, genotyping of SYT11 was analyzed with PCR. C, Mouse melanoma B16F10-luciferase (Luc) cells (2 × 105 cells per mouse) were injected into the tail vein of C57BL/6 mice (9 WT and SYT11-Tg mice). After 18 days, 100 μl of D-luciferin (PerkinElmer, Waltham, MA) was administered intraperitoneally, and luminescence imaging was recorded 10 minutes later. Lung metastasis was measured once a week using the IVIS Lumina II system (Caliper Life Sciences, Hopkinton, MA). Quantification of luciferase activity is presented as photons/sec. (WT: *n* = 9, SYT11 Tg: *n* = 9). **Supplementary Figure 5.** The role of JNK in GC cells. A, The effect of JNK knockdown on GC cell proliferation. Cell viability was analyzed via the SRB assay (*n* = 3). B, JNK expression in GC cells. The JNK protein expression was analyzed with western blotting. C. Cells were treated with JNK inhibitor (SP600125) for 72 h. Viability for cell proliferation was analyzed via the SRB assay (*n* = 4). D. Transwell invasion assay was performing using cells treated with 40 μM SP600125 (*n* = 3). ***p ≤ 0.005 (Student’s t-test).

**Additional file 2: Supplementary Table 1.** Antibody information. **Supplementary Table 2.** List of 45 genes.

## Data Availability

The datasets supporting the conclusions of this article are included within the article and its additional files. The microarray data set of gastric cancer samples from patients is available in the NCBI Database of GEO datasets under the data series accession numbers GSE13861 and GSE84437.

## Abbreviations

ACRG: Asian Cancer Research Group
ANGPTL2: Angiopoietin-like 2
ASO: Antisense oligonucleotide
EMT: Epithelial-mesenchymal transition
FBS: Fetal bovine serum
GC: Gastric cancer
H&E: Hematoxylin and eosin
IHC: Immunohistochemistry
IP: Immunoprecipitation
JAM3: Junctional adhesion molecule 3
JNK: Jun N-terminal kinase
OS: Overall survival
PBS: Phosphate-buffered saline
PCR: Polymerase chain reaction
PI: Propidium iodid
SRB: Sulforhodamine B
SYT11: Synaptotagmin 11
THBS4: Thrombospondin 4
TCGA: The Cancer Genome Atlas
YSH: Yonsei University Severance Hospital
