# Supplementary material for: Synaptotagmin 11 scaffolds MKK7–JNK signaling process to promote stem-like molecular subtype gastric cancer oncogenesis
Source: J Exp Clin Cancer Res. 2022 Jun 29;41:212. doi: 10.1186/s13046-022-02420-3 (PMC9241269; doi:10.1186/s13046-022-02420-3)
Supplement: Supplementary file 1 — Additional file 1: Supplementary Figure 1. Identification of SYT11 as a marker for the stem-like molecular subtype of GC. A and B, Gene Ontology analysis by molecular subtype in GC patients. C, Schematic diagram of in vitro and in vivo RNA interference (RNAi) screening. D, The effect of gene knockdown in stem-like GC cells during the RNAi screen. SNU484 cells were infected with lentiviral shRNAs of each gene for 48 h. Cell viability was analyzed by SRB assay (n = 3). Supplementary Figure 2. Comparison of gene expression by molecular subtype of patients with GC. A, The mRNA expression of SYT7, SYT8, SYT11, and SYT13 in GC patients with intestinal (n = 116) or stem-like (n = 141) molecular subtypes compared with a two-tailed t-test. B, The mRNA expression level of THBS4, Vimentin, JAM3, and ANGPTL2 in GC patients with intestinal molecular subtype (n = 116) or stem-like molecular subtype (n = 141). Supplementary Figure 3. SYT11 expression and cell proliferation after SYT11 knockdown. A, SNU484 cells were treated with 20 nM siSYT11 for 48 h. The mRNA expression of SYT11 and RPL13A was measured with RT-PCR. PSK4 cells were treated with 20 nM siSYT11 for 48 h. The protein expression of SYT11 was analyzed with a western blot. B, SNU484 cells were treated with 20 nM siSYT11 for 48 h. Cell viability was analyzed with the SRB assay (n = 3). Supplementary Figure 4. Effect of SYT11 on lung metastasis in mice. A, SYT11-HA transgenic mice were created by Macrogen (Seoul, Korea). F2 generation male mice were used for the animal study. For human SYT11 genotyping, we used the following primers: F-5′-GTGGATAGCGGTTTGACTCAC-3′ and R-5′-GAAGGTCTCGTCAAACACAGG-3′. Total RNA was extracted from the tissue of WT and SYT11-Tg mice (RNeasy mini kit, Qiagen, Valencia, CA). The mRNA expression of human SYT11 and mouse GAPDH was analyzed with RT-PCR. B, Genotype identification of SYT11 transgenic mouse. Genomic DNA was extracted from the tail of the mouse for genotyping (Wizard genomic DNA purifi [file 13046_2022_2420_MOESM1_ESM.docx]

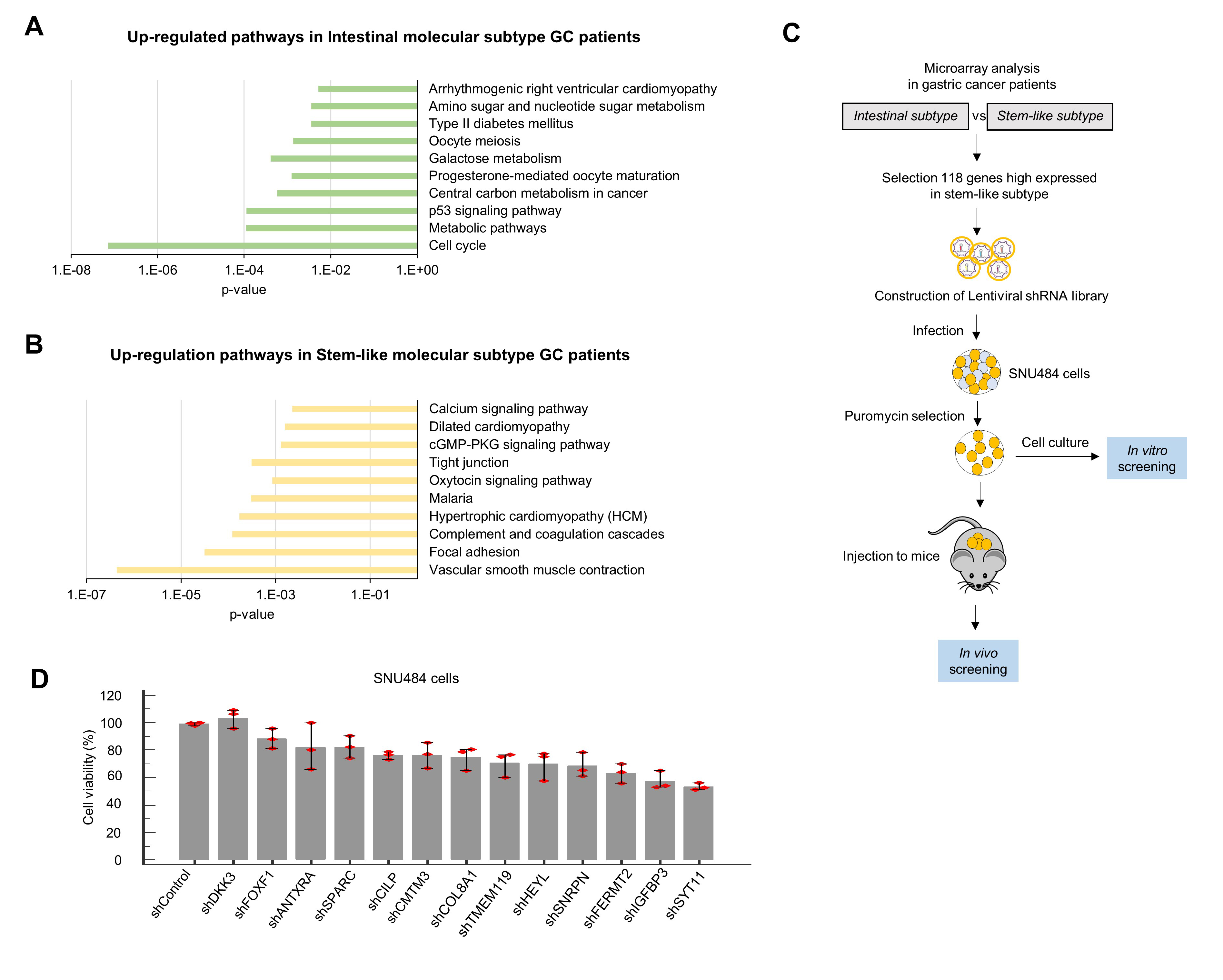


**Supplementary Figure 1.** Identification of SYT11 as a marker for the stem-like molecular subtype of GC. **A** and **B,** Gene Ontology analysis by molecular subtype in GC patients. **C,** Schematic diagram of *in vitro* and *in vivo* RNA interference (RNAi) screening. **D,** The effect of gene knockdown in stem-like GC cells during the RNAi screen. SNU484 cells were infected with lentiviral shRNAs of each gene for 48 h. Cell viability was analyzed by SRB assay (n = 3).


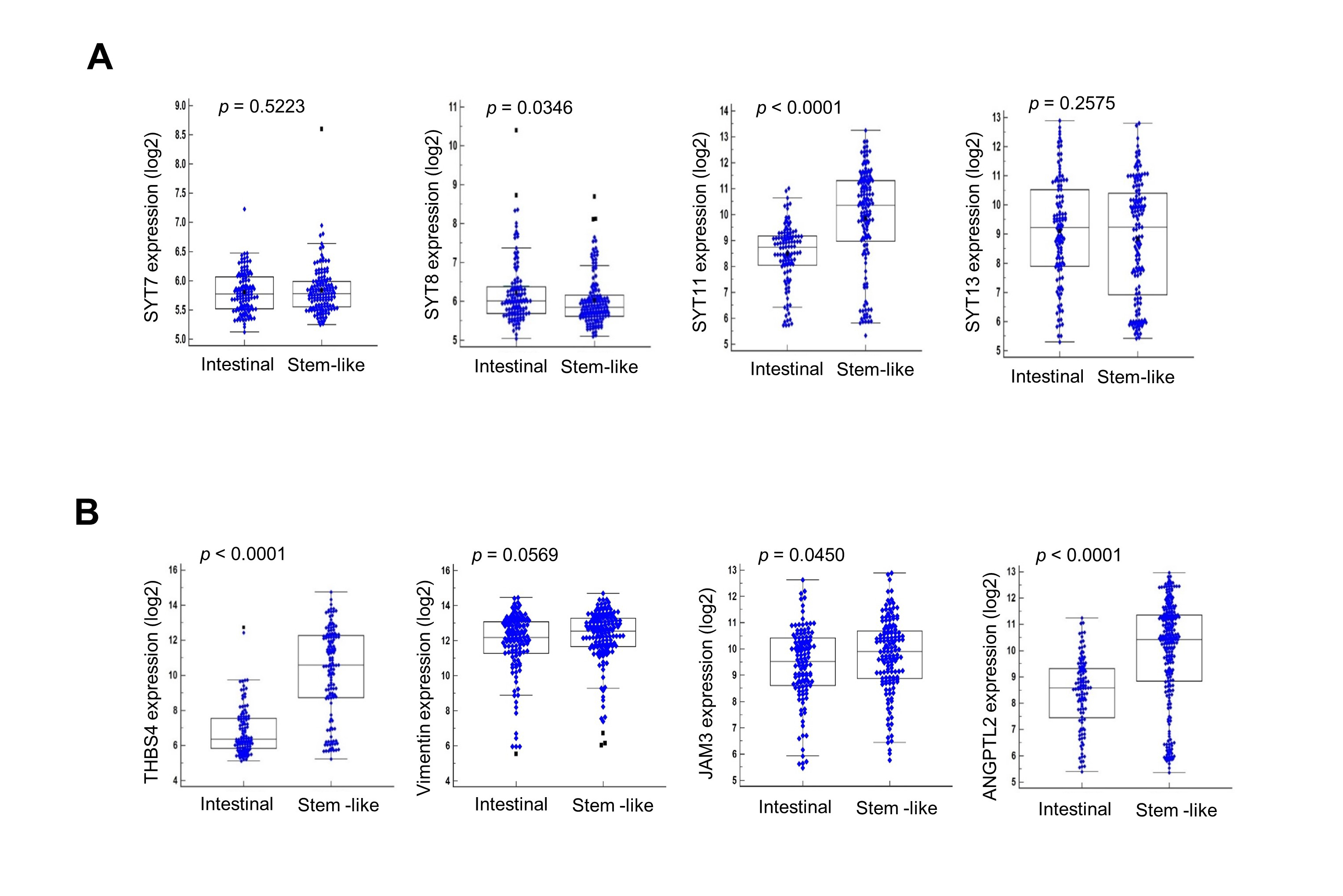


**Supplementary Figure 2.** Comparison of gene expression by molecular subtype of patients with GC. **A,** The mRNA expression of SYT7, SYT8, SYT11, and SYT13 in GC patients with intestinal (n = 116) or stem-like (n = 141) molecular subtypes compared with a two-tailed t-test. **B,** The mRNA expression level of THBS4, Vimentin, JAM3, and ANGPTL2 in GC patients with intestinal molecular subtype (n = 116) or stem-like molecular subtype (n = 141).


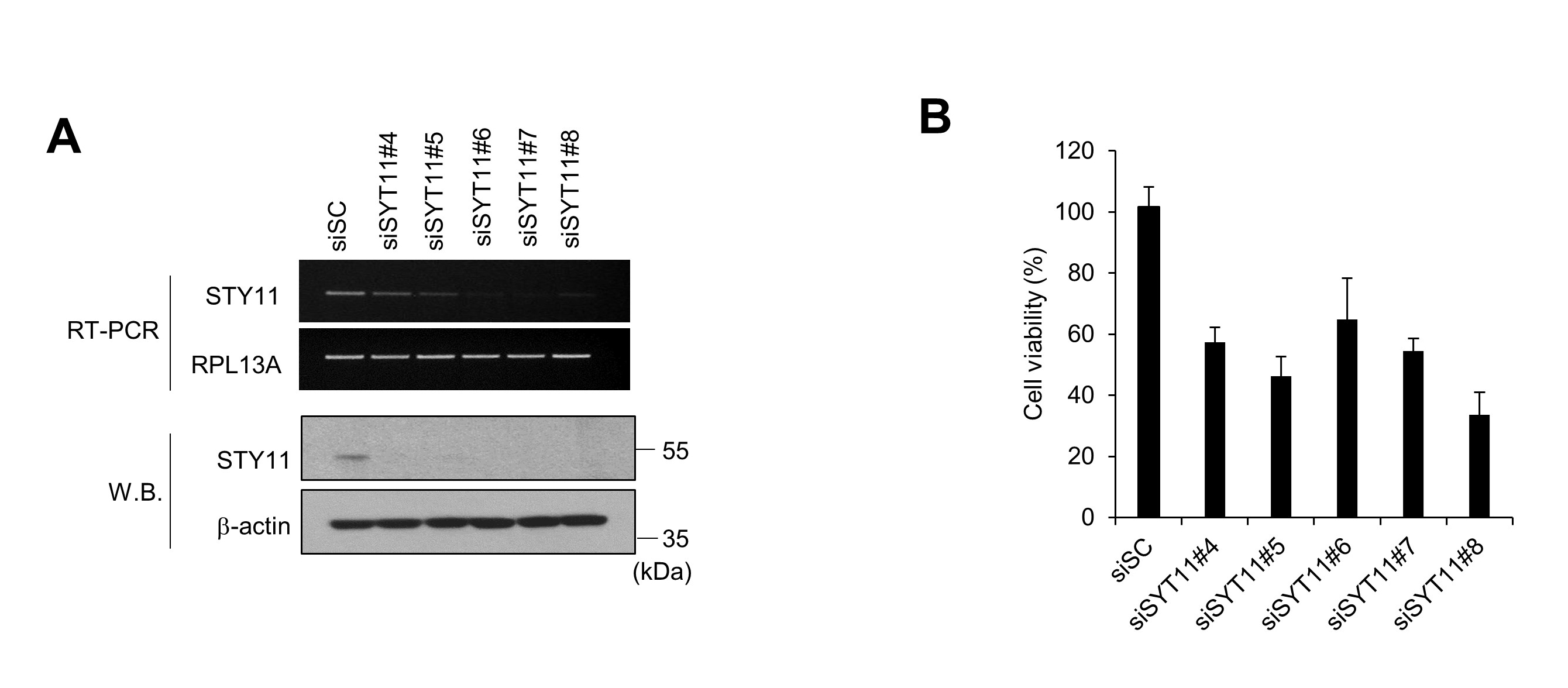


**Supplementary Figure 3.** SYT11 expression and cell proliferation after SYT11 knockdown. **A,** SNU484 cells were treated with 20 nM siSYT11 for 48 h. The mRNA expression of SYT11 and RPL13A was measured with RT-PCR. PSK4 cells were treated with 20 nM siSYT11 for 48 h. The protein expression of SYT11 was analyzed with a western blot. **B,** SNU484 cells were treated with 20 nM siSYT11 for 48 h. Cell viability was analyzed with the SRB assay (n = 3).


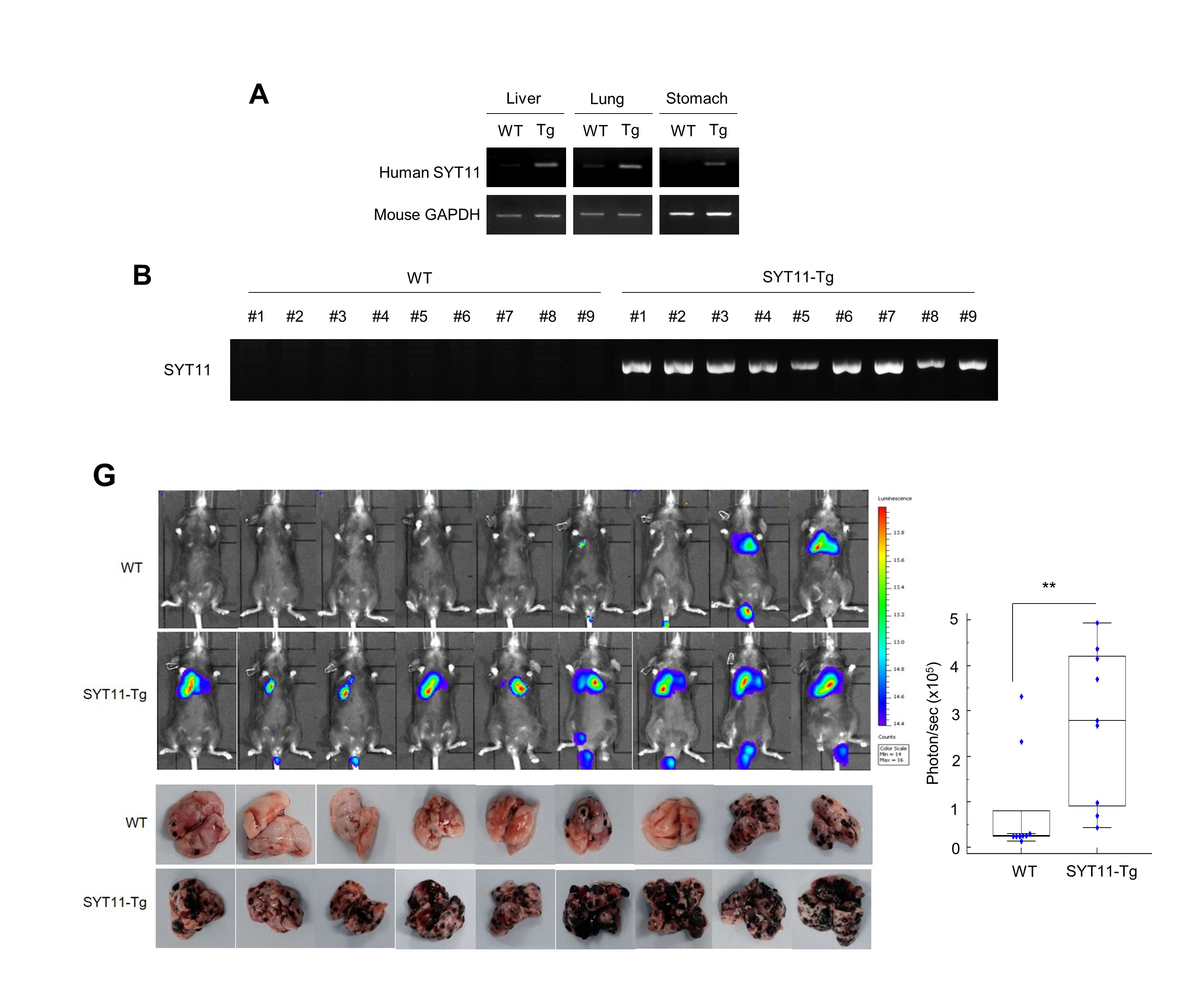


**Supplementary Figure 4.** Effect of SYT11 on lung metastasis in mice. **A,** SYT11-HA transgenic mice were created by Macrogen (Seoul, Korea). F2 generation male mice were used for the animal study. For human SYT11 genotyping, we used the following primers: F-5’-GTGGATAGCGGTTTGACTCAC-3’ and R-5’-GAAGGTCTCGTCAAACACAGG-3’. Total RNA was extracted from the tissue of WT and SYT11-Tg mice (RNeasy mini kit, Qiagen, Valencia, CA). The mRNA expression of human SYT11 and mouse GAPDH was analyzed with RT-PCR. **B,** Genotype identification of SYT11 transgenic mouse. Genomic DNA was extracted from the tail of the mouse for genotyping (Wizard genomic DNA purification kit, Promega Madison, WI). In WT and SYT11-Tg mice, genotyping of SYT11 was analyzed with PCR. **C,** Mouse melanoma B16F10-luciferase (Luc) cells (2 × 105 cells per mouse) were injected into the tail vein of C57BL/6 mice (9 WT and SYT11-Tg mice). After 18 days, 100 μl of D-luciferin (PerkinElmer, Waltham, MA) was administered intraperitoneally, and luminescence imaging was recorded 10 minutes later. Lung metastasis was measured once a week using the IVIS Lumina II system (Caliper Life Sciences, Hopkinton, MA). Quantification of luciferase activity is presented as photons/sec. (WT: n = 9, SYT11 Tg: n = 9).


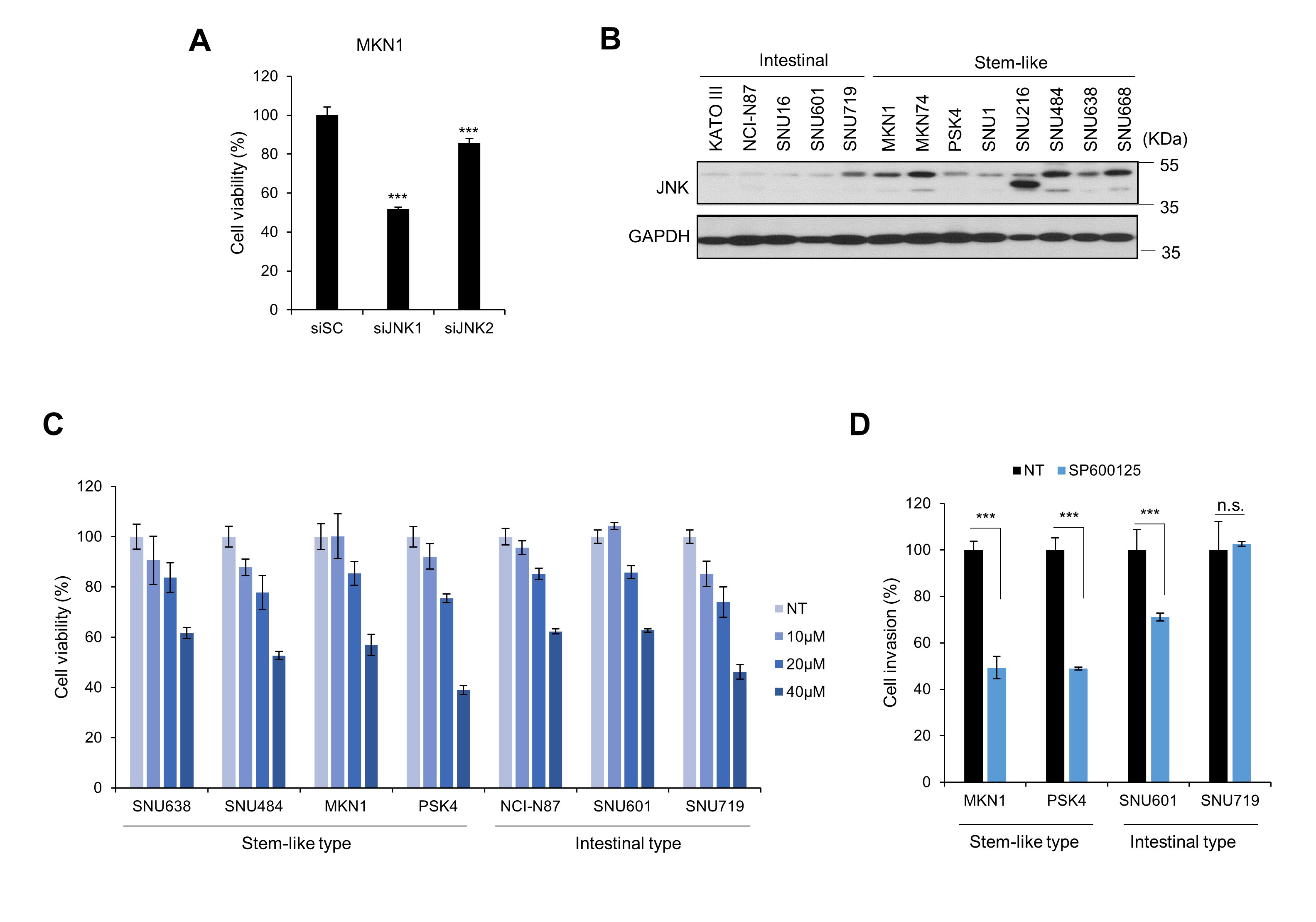


**Supplementary Figure 5.** The role of JNK in GC cells. **A,** The effect of JNK knockdown on GC cell proliferation. Cell viability was analyzed via the SRB assay (n = 3). **B,** JNK expression in GC cells. The JNK protein expression was analyzed with western blotting. C. Cells were treated with JNK inhibitor (SP600125) for 72 h. Viability for cell proliferation was analyzed via the SRB assay (n=4). D. Transwell invasion assay was performing using cells treated with 40 μM SP600125 (n=3). ***p ≤ 0.005 (Student’s t-test).
